# Nanopore-based amplicon sequencing for rapid detection and identification of *Bacillus* spp. in plant-based products

**DOI:** 10.3389/fmicb.2025.1701533

**Published:** 2025-12-19

**Authors:** Marta Bisaschi, Paolo Bellassi, Alessandra Fontana, Maria Luisa Callegari, François Bourdichon, Antonio Del Casale, Fabio Fracchetti, Lorenzo Morelli, Vania Patrone

**Affiliations:** 1 Department for Sustainable Food Process (DiSTAS), Università Cattolica del Sacro Cuore, Piacenza and Cremona, Italy; 2 Microbion S.r.l, Verona, Italy

**Keywords:** amplicon sequencing, *Bacillus* spores, ddPCR, MinION, Oxford Nanopore Technologies, plant-based products, rapid detection, *tuf* gene

## Abstract

*Bacillus* contamination in plant-based food products is a significant concern due to heat-resistant spores that can survive heating and proliferate during storage or handling, possibly leading to foodborne illnesses. Given the potential of high-throughput DNA sequencing to enhance microbial monitoring, we used a targeted approach by combining amplification of the *Bacillus tuf* gene with Oxford Nanopore sequencing. The ability of the MinION-based protocol to detect and identify closely related *Bacillus* species was first assessed using plant-based food samples spiked with spore suspensions of five representative *Bacillus* strains. A DNA extraction method, relying on food enzymatic pre-processing combined with mechanical cell lysis, was implemented to maximize *Bacillus* spore DNA recovery, and a droplet digital PCR (ddPCR) assay was used to evaluate extraction efficiency. Afterwards, the approach was applied to 72 different commercial plant-based foods and supplements to compare nanopore-based *tuf* profiling with established nanopore-based 16S rRNA analysis and culture-based methods. ddPCR analysis showed the high efficiency of the DNA extraction procedure for *Bacillus* spores from spiked samples. Sequencing of the *tuf* gene with the MinION device successfully differentiated the five different *Bacillus* species selected as reference strains for the artificial inoculation of food, when the resulting sequences were aligned against the custom-made BacTufDB database. Tests on commercial products confirmed the *tuf* gene ability (over the 16S rRNA gene) to highlight the presence of *Bacillus* species: *Bacillus* and closely related genera were detected in 35 of the tested plant-based products, out of which seven were contaminated by *Bacillus cereus* group. The study demonstrated that the *tuf*-based methodology more effectively detects *Bacillus* species in plant-based products, offering potential applications in food safety and quality control.

## 1 Introduction

In recent years, increasing attention has been directed toward the microbiological safety of plant-based products, a novel and rapidly expanding category within the food sector. Despite the limited literature on the microbiology of these products, the few studies conducted to date have highlighted that these products may pose significant food safety risks (Wang et al., 2022; Lin et al., 2023). *Bacillus* species are of particular concern in plant-based products due to their natural association with soil and plants, as well as their ability to form heat-resistant spores that can survive common thermal processing methods (Osimani et al., 2021). Previous studies, in fact, already demonstrated the presence of *Bacillus* spp. in plant-based meat analogues (Pernu et al., 2020; Tóth et al., 2021; Hai et al., 2024; Roch et al., 2024); of which 5% belonged to diarrhea-associated strains of the *Bacillus cereus* group (Liu et al., 2017; Makuwa et al., 2023; Tohya et al., 2021; Barmettler et al., 2025). This group is of particular public health concern due to its ability to produce a range of toxins, including enterotoxins responsible for diarrheal syndrome and the emetic toxin *cereulide*, which is associated with severe foodborne intoxications (EFSA BIOHAZ Panel, 2016). Nevertheless, a huge outbreak of *B. cereus* intoxication in plant-based oat drink was recorded in 2022 (Rapid Alert System for Food and Feed, 2022). Furthermore, *B. cereus* and *B. licheniformis* were also shown to be among the most common aerobic spore-forming species in plant-based ingredients used for dairy alternatives (Kyrylenko et al., 2023). Even for plant-based food supplements, *Bacillus* has been shown to be a major contaminant, found in 56% of the tested products (Brown and Jiang, 2008; Dlugaszewska et al., 2019). Thus, pathogenic and spoilage species not only contaminate plant-based products, but certain species have been demonstrated to proliferate at a faster rate in these products compared to dairy products (Tóth et al., 2021; Bartula et al., 2023; Liu et al., 2023).

As emphasized by Lin et al. (2023), it is imperative to develop and implement reliable methodologies for pathogen detection in plant-derived products, to ensure safe consumption and foster sustainable growth in the plant-based food sector. Furthermore, since modern production systems are such intensive and rapid, having methodologies able to cope with their speed and intensity is still a milestone. The aim of this study was, therefore, to provide an approach for rapid, high-throughput targeted sequencing using The Oxford Nanopore Technologies (ONT), to exploit the portability, rapidness, and easiness of the MinION device. ONT has the capability to sequence long and extra-long DNA fragments, resulting in a significant enhancement in the bacterial identification accuracy and reliability (Yang et al., 2020; Wang et al., 2021). Traditionally, 16S rRNA gene sequencing has been the standard for bacterial taxonomic profiling due to its broad applicability across prokaryotic taxa. However, recent studies have highlighted that the *tuf* gene, which encodes elongation factor Tu, displays higher species-level discriminatory power in several bacterial genera, including *Staphylococcus* (McMurray et al., 2016), *Lactococcu*s (Pourahmad, 2025), *Acetobacter* (Huang et al., 2014), *Enterococcus* (Li et al., 2012), and *Lactobacillus* (Naser et al., 2007). Specifically, for the *Bacillus subtilis group*, *tuf*-based identification has proven more accurate than 16S rRNA-based methods (Xu et al., 2023). Based on this evidence, we evaluated the combination of the *tuf* gene sequencing with Oxford Nanopore technology to assess its potential for detecting *Bacillus* in plant-based products. We hypothesized that long-read ONT sequencing based on the *tuf* gene could provide higher discriminatory power than conventional 16S rRNA gene sequencing for identifying *Bacillus* species in complex food matrices. To achieve this objective, we first implemented a direct DNA extraction method for *Bacillus* spores in plant-based products, followed by the development of a protocol for long-read *tuf* amplicon sequencing using a MinION device.

As proof of concept, the ability of the sequencing protocol to properly identify relevant, closely related *Bacillus* species was initially assessed in food samples spiked with spores of different *Bacillus* reference strains. The methodology was then validated using 72 different commercially available plant-based products and supplements, and the results were compared with those obtained through 16S rRNA gene sequencing and culture-based analyses. Overall, the implemented molecular approach could represent a straightforward analytical solution for the detection of *Bacillus* spp. and closely related genera in complex plant-based food matrices, potentially contributing to food safety monitoring in an increasingly relevant sector of the food industry.

## 2 Materials and methods

### 2.1 Preparation of samples

#### 2.1.1 *Bacillus* spore preparation and mock DNA extraction

Five bacterial representative strains were purchased from the German Collection of Microorganisms and Cell Cultures (DSMZ) and used as marker microorganisms for inoculation experiments of plant-based products: *Bacillus cereus* ATCC 14579^T^, *Bacillus thuringiensis* ATCC 10792^T^, *Bacillus licheniformis* ATCC 14580^T^, *Bacillus subtilis* ATCC 6051^T^, and *Bacillus atrophaeus* ATCC 9372^™^. The latter is utilized as a biological indicator of sterilization treatments to validate and monitor the efficacy of sanitization processes (FDA, 2007). Vegetative cells were grown on Nutrient Agar (Sigma – Aldrich, Milan, Italy) plates, overnight, at 30 °C. For sporulation, bacterial strains were cultivated in 250 mL of Difco sporulation medium (DSM), as described by Nicholson and Setlow (1990). Cultures were incubated at 30 °C shaking at 200 rpm, for 72 h (*B. subtilis, B. licheniformis* and *B. atrophaeus*) or for 10 days (*B. thuringiensis* and *B. cereus*). The extent of sporulation was determined by examination of cultures using phase-contrast microscopy, as described by Nicholson and Setlow (1990). Spores were harvested when the percentage of phase-bright spores to vegetative cells reached ≥ 90%, as determined using a Thoma chamber. After centrifugation at 14,000 × g for 20 min, the supernatant was discarded, leaving 30 mL of broth to resuspend spores collected in the pellet. The suspension was then incubated overnight at 37 °C with lysozyme (50 μg mL^−1^; Sigma – Aldrich, Milan, Italy). Spore purification was performed by density gradient centrifugation (8,000 × g for 30 min) using Percoll solution (Sigma – Aldrich, Milan, Italy) at different concentrations (with density = 1.13; 1.11; 1.09; 1.07; 1.06). Finally, purified spore solutions were washed five times with sterile water (each washing step was performed by centrifugation at 14,000 × g for 20 min at 4 °C to pellet the spores before the subsequent resuspension in sterile distilled water), and their concentration was determined by direct counting using a Thoma chamber. Purified spores were stored in sterile distilled water at 4 °C until use, according to the procedure described by Nicholson and Setlow (1990). DNA extractions of purified spores of all five *Bacillus* species were conducted on 1 mL of 10^6^ spores × mL^−1^ solution, using the Master Pure Gram-Positive DNA purification kit (LGC Biosearch Technologies). DNA yield and integrity were verified by quantification with Qubit dsDNA HS assay kit (Invitrogen, Carlsbad, CA) and by gel electrophoresis on an Agilent 4500 Tape station using D5000 High Sensitivity amplicon kit (Agilent Technologies, Santa Clara, CA). An equimolar pool of metagenomic DNA of the five target *Bacillus* strains was used as a mock community.

#### 2.1.2 Artificial contamination with spores, matrix pre-treatment and DNA extraction

**Figure 1:**
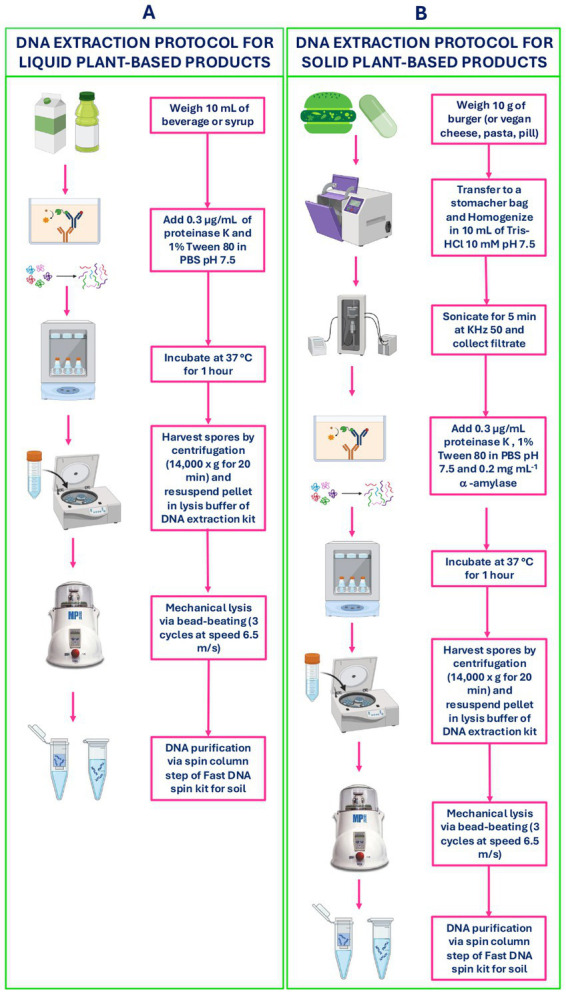
Workflow of the DNA extraction procedures for *Bacillus* spores from **(A)** liquid (beverages and syrups) and **(B)** solid (burgers, vegan cheese, vegan pasta, and pills) plant-based products.

An ultra-high temperature (UHT) soy beverage, a soy burger, a soy cheese, a vegan pasta, a plant-based syrup, an herbal pill and an herbal tea infusion (packed food products, purchased at local supermarkets) were used as representative samples of either liquid or solid plant-based foods and supplements products. Aliquots of each food sample were spiked with a mixed suspension of spores from the five bacterial representative strains in the same proportions, to achieve final concentrations from 10^1^ to 10^5^ spores × mL^−1^ (or g^−1^ for solid products). For liquid products, spores were directly added and mixed into 10 mL of product, whereas for solid products, 10 g were first diluted and homogenized in a stomacher bag in 10 mL of 9% Tris–HCl solution before spiking. For all solid products, also, a 5-min 50 KHz treatment in an ultrasonic bath (Falc Instruments, Treviglio, Italy) was performed to help release spores from solid food particles (De Boer et al., 2015). Moreover, samples were passed through the filter of the stomacher bag to remove large food particles. To maximize the recovery of spores from the food matrix, both liquid and solid samples were digested with 0.3 μg mL^−1^ of proteinase K (Sigma – Aldrich, Rome, Italy) and 1% Tween 80^®^ (Sigma – Aldrich, Rome, Italy), directly added to the filtered product and incubated at 37 °C for 1 h to resolve proteins and fat. In addition, for burger samples significantly rich in starch, 0.2 mg mL^−1^ of α-amylase (Sigma – Aldrich, Rome, Italy) were also used, with an incubation of 65 °C for 6 min. Each step of the pre-processing process, including optimization of enzymes concentration, incubation conditions and the optional use of an ultrasonic bath, was evaluated by plating aliquots of the matrix before and after each step on Tryptic Soy Agar plates (TSA) (Sigma – Aldrich, Rome, Italy) and counting the recovered spores. This allowed us to quantify the number of spores released at each stage and assess the efficiency of spore recovery from the different food matrices. Samples were then centrifuged at 14,000 × g for 15 min, and DNA was extracted from the pellet using the Fast DNA Spin kit for soil (MP Biomedicals) according to manufacturer’s instructions, but with an increase in bead beating mechanical lysis: Three cycles of bead beating with FastPrep-24 instrument (60 s each cycle at the maximum speed of 6.5 m × s^−1^) were performed to yield the highest amount of DNA from spores, with minimal DNA degradation (Esteban et al., 2020). A schematic representation of the extraction protocol is shown in Figure 1.

#### 2.1.3 Evaluation of DNA extraction efficiency by ddPCR

To check the performance of the DNA extraction method applied to artificially contaminated products, a ddPCR analysis was performed. A probe assay targeting *B. cereus*, *B. subtilis* and *B. licheniformis*, and composed by forward primer (5′-CCTACGGGAGGCAGCAGTAG-3′), reverse primer (5′-GCGTTGCTCCGTCAGACTTT-3′) and a probe (5′-FAM-AATCTTCCGCAATGGA-MGB-3′) (Fernández-No et al., 2011), was used to amplify a portion of the 16S rRNA gene of the five target *Bacillus* species (an *in silico* PCR was previously performed to check that the primer set could also amplify *B. thuringiensis* and *B. atrophaeus*).^1^ Negative controls, consisting of DNA extracted from food samples that were not artificially contaminated (“non-inoculated samples”), were run to exclude indigenous *Bacillus* spp., while “positive controls” (consisting of the DNA extracted from the five *Bacillus* strains) were used to verify that the assay could efficiently amplify all target strains. No template controls were included. Sample dilutions from 1:10 to 1:10,000 were tested. DNA amplification was conducted in a C1000 Touch Thermal cycler (Bio-Rad, Hercules, CA) with the annealing temperature lowered by 2 °C compared to the original protocol by Fernandez et al.: DNA amplification was carried out with an initial activation at 95 °C for 10 min, followed by 40 cycles of 95 °C for 15 s and 58 °C for 60 s, and a final step at 98 °C for 10 min. The composition of the PCR reaction mixture was as follows: 11 μL of ddPCR No dUTP Supermix for Probes (BioRad, Hercules, CA), 2 μL of template DNA, 900 nM of assay primer+probe (in 3.6:1 ratio, respectively) (BioRad, Hercules, CA) and 8 μL of nuclease free water to reach up the final reaction volume of 22 μL. After DNA amplification, the droplets were read in a QX600 Droplet Digital PCR System (Biorad, Hercules, CA), and the results were analyzed with QuantaSoft^™^ software, version 1.7 (Biorad, Hercules, CA). For precise and reliable target quantification, a cut-off of 10,000 droplets was set. The obtained number of gene copies was converted to the number of Log_10_ equivalent genomes × mL^−1^ (or g^−1^) of product, considering 10.5 as an average number of copies of the 16S rRNA gene in the genome of the five inoculated strains.^2^

### 2.2 ONT amplicon sequencing

#### 2.2.1 Amplification of *tuf* and 16S rRNA genes

A modified set of primers, based on those designed by Caamaño-Antelo et al. (2015), was used for the amplification of a 791 bp segment of the *Bacillus* spp. *tuf* gene: the modified forward primer m-tufGPF (5′-ACGTTGACTGCCCAGGHCAC-3′) and the reverse primer tufGPR (5′-GATACCAGTTACGTCAGTTGTACGGA-3′). The insertion of the degeneration in the forward one was made as a result of an *in silico* analysis, carried out with ClustalW sequences alignment tool (Larkin et al., 2007). Specifically, *tuf* gene sequences of 1,438 strains belonging to 125 *Bacillus* spp. were extracted from the NCBI database (considering complete and reference genomes only; Supplementary Table S1) and aligned. Graphical representation of primer sequences, based on the sequence alignments, was carried out with WebLogo,^3^ showing conserved (i.e., > 90% nucleotide identity) and variable regions of the *tuf* gene. Furthermore, to investigate m-tufGP primer specificity, *in silico* PCR was performed using the “Test Primers” tool of Geneious Prime software (version 2020.1.2) (Kearse et al., 2012) against the BacTufDB database (see paragraph 2.2.2). Amplification was evaluated allowing up to three mismatches per primer, and performance was assessed using the following metrics: sensitivity (fraction of *Bacillus* sequences correctly amplified), specificity (fraction of non-*Bacillus* sequences correctly excluded), precision, expressed as Positive Predictive Value—PPV (fraction of amplified sequences belonging to *Bacillus*), and accuracy (overall fraction of correctly classified sequences). The formulas used for these calculations are provided in Supplementary Table S2.

Amplification of *tuf* gene by polymerase chain reaction (PCR) was performed in a Mastercycler EP Thermal Cycler (Eppendorf). Each section contained 12.5 μL of Kapa High Fidelity PCR Master Mix 2X (Roche Diagnostics), 2.5 μL of each 10 μM nucleotide primer and 7.5 μL of template DNA. The amplification conditions were as follows: initial denaturation at 95 °C for 5 min followed by 35 cycles each with denaturation at 98 °C for 25 s, annealing at 60 °C for 20 s, and extension at 72 °C for 30 s, being followed by a final elongation step at 72 °C for 5 min.

16S rRNA gene amplification was carried out using the universal primer pair 27F (5′-AGRGTTYGATYHTGGCTCAG-3′) and 1492R (5′-RGYTACCTTGTTACGACTT-3′) under the following PCR conditions: 95 °C for 5 min; 20 cycles of 98 °C for 20 s, 55 °C for 45 s, and 72 °C for 3 min; and 72 °C for 5 min. For the reaction mix, 12.5 μL of Kapa High Fidelity PCR Master Mix 2X (Roche Diagnostics, Basilea, Switzerland) were added with 2.5 μL of each 10 μM nucleotide primer. PCR products were checked on a 1% agarose gel and purified with the 1.8x Ampure XP of Agencourt AMPure XP purification kit (Beckman Coulter, Brea, CA). To obtain sufficient PCR product for sequencing, samples spiked with a concentration of 10^2^ spores × mL^−1^ (or g^−1^) were amplified in triplicates and amplicons were merged and concentrated on spin filter columns (NucleoSpin Gel and PCR Clean-up kit, Macherey Nagel, Dueren, Germany). DNA amplicons were quantified using a Qubit fluorometer and Qubit dsDNA HS assay kit (Invitrogen, Carlsbad, CA) according to the manufacturer’s instruction. The quality and integrity of amplicons DNA were determined by the NanoDrop spectrophotometer (ThermoFisher Scientific, Waltham, MA) and by gel electrophoresis on an Agilent 4,500 Tapestation using D5000 High Sensitivity amplicon kit (Agilent Technologies, Santa Clara, CA).

#### 2.2.2 Library preparation and sequencing, *tuf* custom-database creation and bioinformatic analyses

Libraries were prepared following the standard protocol for the Native Barcoding Kit 24 v14 (SQK-NBD114.24, ONT) for sequencing on MinION device and checked with Agilent 4,500 Tapestation using D5000 High Sensitivity amplicon kit (Agilent Technologies, Santa Clara, CA) before loading 50 fmol of library on a MinION R10.4.1 flow cell (FLO-MIN114, ONT). Sequencing runs were performed for 8–12 h to process all 24 barcoded samples, depending on the number of active pores available at the start of the run.

Data acquisition and streaming, feedback runs, demultiplexing, and adapter cutting were performed using MinKNOW (v23.11.5). Raw signal data were stored in POD5 format and were converted to FASTQ files with the super accuracy (SUP) basecalling performed by Dorado (v0.5.3) (ONT-developed software) using a standard laptop workstation hardware equipped with GPU NVIDIA GeForce RTX 3080 with 16 GB of RAM, ensuring efficient and accurate processing of raw signal data. Metrics including number and quality of reads obtained after basecalling were investigated using the NANOPLOT tool.^4^ Passed FASTQ files were processed in the EPI2ME Labs platform (v5.1.10) for onward analyses. Reads were filtered based on quality (only those with a Q-score >10 were considered) and length (700–1,000 bp for the *tuf* gene and 1,200–1,600 bp for the 16S rRNA gene). Then, taxonomic classification was made using the embedded alignment-based Minimap2 tool (v2.30) (Li, 2018) with default parameters (minimum identity = 90%; minimum coverage = 0%) and a closed-reference mapping approach, using the desktop workstation equipped with 10 CPU cores, 20 threads, and 256 GB of RAM.

A *tuf*-gene custom database (henceforth referred to as BacTufDB) was built from all *tuf* genes extracted from the genome collection on the NCBI database (accessed in May 2024; Supplementary Table S3). Only complete and reference genome sequences were considered to provide a more consistent and reliable classification, for a total of 45,963 genomic sequences, corresponding to 17,419 bacterial species. For 16S rRNA gene analysis, the built-in NCBI 16S/18S SSU database, available within the EPI2ME Labs platform, was used. The Bray–Curtis similarity index was calculated using PAST software (v4.17). This index measures the similarity between two samples based on their relative abundances. It is defined by the equation:


$$\mathrm{BC}=1-\frac{\sum_{i=1}^{S}|A_{i}-B_{i}|}{\sum_{i=1}^{S}(A_{i}-B_{i})}$$

Bray–Curtis similarity index was used as a measure for assessing the difference between the actual and expected profiles of the communities, considering the sequenced mock and artificially contaminated samples. The similarity index ranges between 0 and 1, where 1 means that the two communities compared have the same distribution, and 0 means the two consortia are not consistent for species distribution (Bray and Curtis, 1957). To avoid variation due to uneven sequencing effort between samples, a rarefaction step to 10,000 sequences per sample was performed prior to data analysis, with R (ver. 4.2.2) and RStudio (ver. 1.4.1106) (R Core Team, 2021). The rarefaction threshold was chosen based on the Shannon alpha-rarefaction curve to maximize sample retention while maintaining adequate sequencing depth for reliable diversity estimates (Supplementary Figure S1). To assess *Bacillus* and closely related genera community diversity, all the genera other than *Bacillus* were grouped into a single feature (“Others”). To estimate alpha diversity, the Shannon index was computed using R package vegan (version 2.6–10) (Oksanen et al., 2020). Differences in alpha diversity and in the cumulative relative abundance of *Bacillus* and closely related genera, obtained from the two ONT-sequencing approaches, were evaluated using the Wilcoxon signed-rank test, a non-parametric method suitable for paired data without assuming a normal distribution. All results were visualized using GraphPad Prism (version 8.00). To compare the taxonomic identification results between ONT- and culture-based analyses, graphical representations were generated using Intervene tool (Khan and Mathelier, 2017).

### 2.3 Commercial samples

#### 2.3.1 Product description, DNA extraction and ONT sequencing of commercial products

The DNA extraction method (Figure 1) and the PCR (of both *tuf* and 16S rRNA genes) were applied to a total of 72 commercially collected plant-based samples across different categories (in detail: 17 plant-based beverages, 17 meat and fish-analogues, 7 cheese and egg-analogues, 4 vegan ready-to-eat pasta, and 27 plant-origin food supplements, of which 5 liquids and 22 solids). Supplementary Table S4 summarizes the main characteristics of the collected products. In addition to belonging to different types of food, products were quite diverse in terms of composition (protein or plant source), heat treatment (i.e., UHT, pasteurization), and formulation (i.e., pills, syrups, capsules, opercula). Commercial samples that showed an intense and distinct amplification band were further processed for *tuf* and 16S rRNA gene ONT sequencing, while the remaining samples were analyzed by ddPCR, as described in Section 2.1.3, to verify and, if present, quantify *Bacillus* DNA.

#### 2.3.2 Culture-based analyses

Commercial plant-based products were tested by a culture-dependent approach to verify the presence of *Bacillus* species in the tested products. A 10 mL (or 10 g for solids) of products were added to 150 mL of Tryptic Soy Broth (TSB) (Sigma – Aldrich, Rome, Italy) in glass flasks, which were incubated at 30 °C and at 55 °C overnight. A series of decimal dilutions were then prepared and spread on Bacillus Cereus Agar Base plates (PEMBA) (Sigma – Aldrich, Rome, Italy), added with egg yolk and incubated at 30 °C and at 55 °C. For each sample, isolated colonies with different morphologies were picked, re-streaked for purity, and DNA extracted using MicroLYSIS PLUS (Microzone, Stourbridge, UK). The 27F and 1492R primers mentioned above were used to amplify the partial 16S rRNA gene for Sanger sequencing. Sequences identification was carried out with EZBioCloud (Chalita et al., 2024), considering the “*Top-hit taxon*” output.

## 3 Results

### 3.1 DNA extraction efficiency measured by ddPCR

To check the performance of DNA extraction methods, a ddPCR analysis was performed on seven artificially contaminated samples. The positive control consisting of DNA extracted from the five reference *Bacillus* spp. strains was correctly amplified and quantified for each strain (data not shown), and no amplification was observed in any of the no-template controls. For the non-inoculated samples, while for the soy drink, the burger and the syrup a negligible presence of indigenous *Bacillus* spp. was detected, for the vegan cheese, the vegan pasta, the pills and the herbal tea infusion it is substantial (Table 1).

**Table 1:** DNA quantification by ddPCR, expressed as number of equivalent genomes per mL or per g of product, for artificially contaminated samples.

Contamination level (Log_10_ spores/mL or g)	Soy drink	Soy burger	Raspberry syrup	Herbal pills	Vegan soy cheese	Vegan pea pasta	Herbal tea infusion
	Log_10_ equivalent genomes^**^/mL						
–^*^	0.37 ± 0.01	0.69 ± 0.01	0.021 ± 0.01	1.26 ± 0.01	2.31 ± 0.02	2.68 ± 0.02	2.19 ± 0.02
1.00	1.05 ± 0.01	1.17 ± 0.01	1.16 ± 0.02	1.72 ± 0.02	2.34 ± 0.02	2.87 ± 0.02	2.27 ± 0.02
2.00	2.28 ± 0.02	1.90 ± 0.03	2.34 ± 0.01	2.54 ± 0.01	2.48 ± 0.02	2.97 ± 0.02	2.65 ± 0.04
3.00	3.45 ± 0.02	2.74 ± 0.02	3.49 ± 0.02	2.98 ± 0.01	3.05 ± 0.04	3.21 ± 0.04	2.93 ± 0.04
4.00	4.35 ± 0.03	3.79 ± 0.03	4.42 ± 0.02	3.64 ± 0.02	4.01 ± 0.04	4.07 ± 0.04	3.56 ± 0.04
5.00	5.17 ± 0.03	4.87 ± 0.03	5.00 ± 0.03	4.88 ± 0.03	4.97 ± 0.04	5.02 ± 0.04	4.66 ± 0.04
R-squared (R^2^)^***^	0.99	0.97	0.99	0.96	0.94	0.92	0.92

^*^ Non-inoculated samples. ^**^Log_10_ equivalent genomes are calculated from the number of gene copies considering 10.5 as an average number of the 16S rRNA gene copies in the genome of the five inoculated strains. ^***^Coefficient of determination (R^2^) by comparison of inoculated spores and concentration results from ddPCR.

All quantitative results obtained from spiked products are summarized in Table 1. By means of the implemented DNA extraction protocols, ddPCR was able to detect spore DNA over the full range of spore inoculum concentrations. In addition, a correlation between the actual concentrations of artificially contaminated *Bacillus* spores and the values measured by ddPCR across all tested products was observed, as indicated by the coefficient of determination (R^2^). Also, standard deviations calculated among replicates are reported in Table 1.

The soy drink, the burger, the syrup, and the pills were then processed as model artificially contaminated food matrices for ONT sequencing detection.

### 3.2 ONT amplicon sequencing

#### 3.2.1 Mock and artificially contaminated samples

From the alignment results of tufGP primers on *Bacillus* spp. *tuf* genes, the presence of three mismatches was evidenced at the binding site of the forward primer (Supplementary Figure S2): 5 T/A (93.5%/6.5%), 17A/T (69.1%/30.9%), and 20C/T (94.5%/5.5%). Due to the higher frequency of the position 17 mismatch among *Bacillus* spp. (Supplementary Table S5), this degeneration has been selected to proceed with *tuf*-based amplicon sequencing (m-TufGP primers, see methods Section 2.2.1). The primer performance analysis showed that, at a tolerance of ≤ 2 mismatches per primer, the *tuf* primers achieved 98.5% sensitivity, 95.8% specificity, and 96.0% accuracy. When the tolerance was increased to ≤ 3 mismatches per primer, sensitivity rose to 99.2%, while specificity decreased to 73.3%, resulting in an overall accuracy of 74.1%. Detailed results for all mismatch thresholds are provided in Supplementary Table S2.

**Figure 2:**
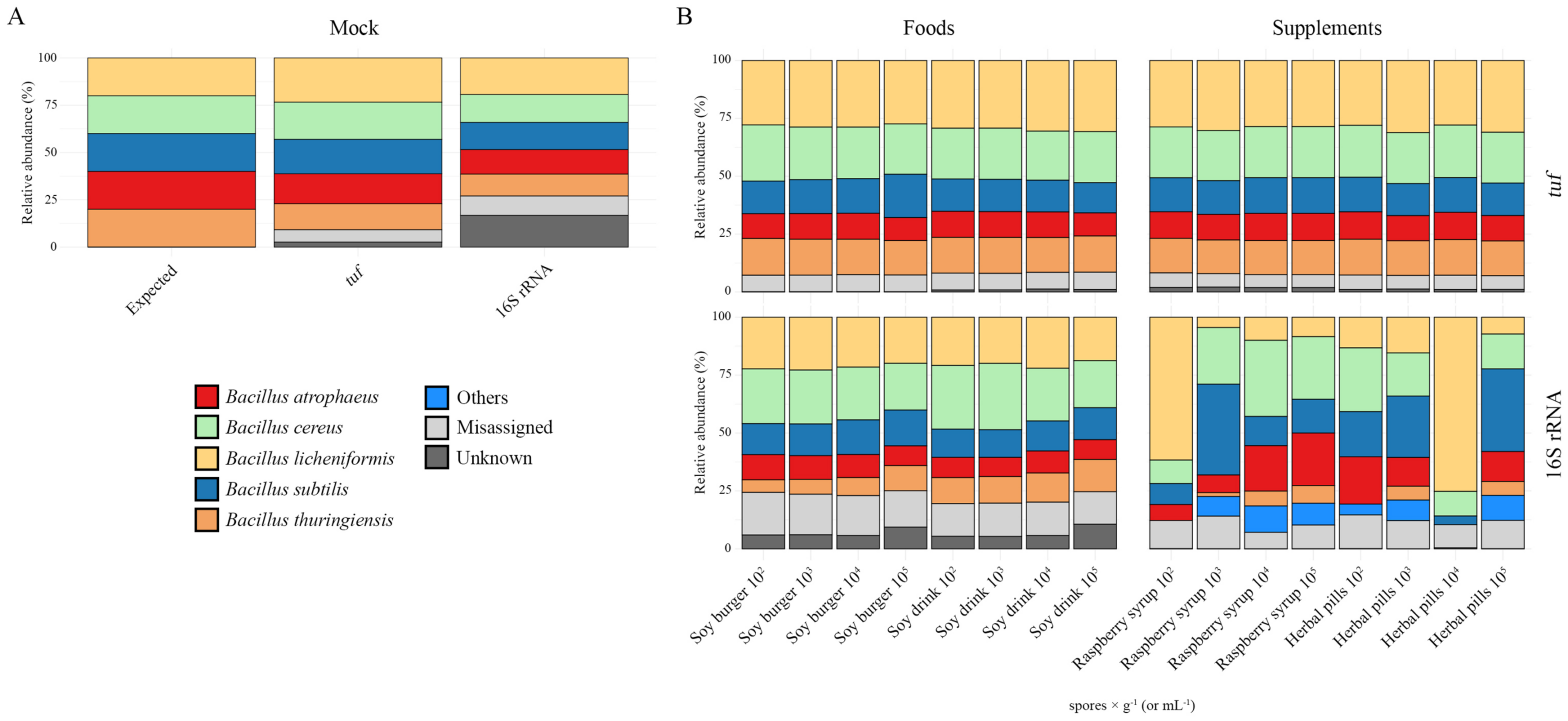
Stacked bar charts representing the ONT-based species identification by *tuf* and 16S rRNA genes of **(A)** the mock community and **(B)** the artificially contaminated samples. All the identified taxa whose relative abundance is lower than 2.5% are collapsed into the “Misassigned” group; unclassified species were grouped as “Unknown,” whereas classified species (different from the mock) with relative abundance higher than 2.5% are collapsed into the “Others” group.

As proof of concept, ONT sequencing was firstly conducted on a simple mock community and on artificially contaminated samples, to elucidate the ability of the MinION-based *tuf* gene sequencing to identify and distinguish pathogenic *Bacillus* species from non-pathogenic associated with plant-based products. Figure 2A compares expected vs. observed abundances in the *Bacillus* mock community, using both *tuf* and 16S rRNA gene sequencing. *Tuf* sequencing across the three replicates generated an average of 119,586 reads per sample and successfully identified all five target species. Details about the statistics of reads after the SUP basecalling, and before and after the quality filtering, for each sample, are reported in Supplementary Table S6. The relative abundances were: 13.45% *Bacillus thuringiensis*, 23.39% *Bacillus licheniformis*, 19.94% *Bacillus cereus*, 18.16% *Bacillus subtilis* and 15.84% *Bacillus atrophaeus,* with 6.59% of reads belonging to misassigned species (none exceeding 2.5% individually) and 2.63% of unassigned. Since the frequency of the most abundant false-positive taxon was below 2.5%, a threshold of relative abundance at 2.5% was set to distinguish background noise in the subsequent analyses. The Bray–Curtis similarity value (Table 2) for the *tuf*-based mock community was 0.87, indicating a well-balanced distribution of the strains and agreement with the expected composition.

**Table 2:** Bray–Curtis similarity index calculated on average values, indicating the similarity between the expected and the ONT-detected relative abundances.

Bray–Curtis index	ONT-*tuf* vs. expected	ONT-16S rRNA vs. expected
Expected	1.00	1.00
Mock	0.87	0.73
Soy burger (10^2^ spores × g^−1^)	0.81	0.70
Soy burger (10^3^ spores × g^−1^)	0.81	0.70
Soy burger (10^4^ spores × g^−1^)	0.81	0.73
Soy burger (10^5^ spores × g^−1^)	0.84	0.75
Soy drink (10^2^ spores × mL^−1^)	0.81	0.72
Soy drink (10^3^ spores × mL^−1^)	0.81	0.72
Soy drink (10^4^ spores × mL^−1^)	0.80	0.75
Soy drink (10^5^ spores × mL^−1^)	0.79	0.75
Raspberry syrup (10^2^ spores × mL^−1^)	0.81	0.46
Raspberry syrup (10^3^ spores × mL^−1^)	0.80	0.54
Raspberry syrup (10^4^ spores × mL^−1^)	0.82	0.69
Raspberry syrup (10^5^ spores × mL^−1^)	0.82	0.71
Herbal pills (10^2^ spores × g^−1^)	0.82	0.73
Herbal pills (10^3^ spores × g^−1^)	0.80	0.72
Herbal pills (10^4^ spores × g^−1^)	0.82	0.34
Herbal pills (10^5^ spores × g^−1^)	0.80	0.61

Based on the DNA amount needed by ONT for library preparations, we determined that samples with a minimum artificial contamination level of 10^2^ spores × mL^−1^ could be subsequently sequenced. For samples with low spore concentrations (10^2^ spores × mL^−1^), three independent PCR amplifications were performed, and the resulting amplicons were pooled prior to sequencing to ensure sufficient DNA yield and representativeness. *Tuf* gene sequencing yielded an average number of 142,574 reads for inoculated plant-based food samples and 235,057 for plant-based food supplements (details about the reads statistics are reported in Supplementary Table S6). A successful resolution of all target species was achieved, with actual proportions of each microorganism reflecting expected relative abundances (Figure 2B). The five target *Bacillus* species accounted the 91% of the total composition in all products. Bray–Curtis similarity values (Table 2) averaged 0.80–0.82 across products, and unassigned sequences remained low (0.09–1.98%), while misassignments ranged from 5.84 to 7.25%.

In parallel, full-length 16S rRNA sequencing was performed for comparison. The mock community generated an average of 91,910 reads per sample, also identifying all five targets, but with lower accuracy: 10.22% of reads were misassigned and 15.22% were unknown, resulting in a Bray–Curtis score of 0.73. Sequences were attributed as follows: 11.59% *Bacillus thuringiensis*, 19.35% *Bacillus licheniformis*, 14.75% *Bacillus cereus*, 14.36% *Bacillus subtilis,* and 12.93% *Bacillus atrophaeus* (Figure 2A) (reads statistics are available in Supplementary Table S7). Since the frequency of the most abundant false-positive taxon was below 2.5%, as for the *tuf* gene analysis, a threshold of relative abundance at 2.5% was determined for distinguishing potential contaminants from background in ONT-based *tuf* sequencing of commercial food products.

For the artificially contaminated samples, read counts averaged 45,069 for food and 163,900 for supplements (statistics of reads are reported in Supplementary Table S7). Although all five targets were detected (Figure 2B), they represented only ~70% of the total community. An additional ~8% consisted of non-target *Bacillus* species, especially in supplements. Consequently, 16S rRNA sequencing of plant-based supplements revealed a less-balanced distribution of the five target *Bacillus* species. Misassigned rates were higher than those observed with *tuf*, reaching up to 17.4% in the soy burger, while those of unknown were lower than with *tuf* gene: 6.76% for soy drink, 6.77% for soy burger, 0.04% for syrup, and 0.3% for pills. In soy drink and burger samples, the rate of unknown progressively increased from 10^2^ spores × mL^−1^ to 10^5^ spores × mL^−1^ contamination level. Bray–Curtis similarity values (Table 2) for ONT-based 16S rRNA for the artificially contaminated samples were generally lower, with values of 0.72 for soy drink and burger, 0.60 for syrup and pills.

#### 3.2.2 ONT analyses of plant-based commercial products

For the *tuf* gene, 40 of the 72 commercial products tested yielded visible, discrete bands of the expected size (Supplementary Figure S3) and were subsequently processed for sequencing. 16S rRNA analysis was performed for comparison. The other 32 products (out of the 72 tested) that did not show any amplification band suitable for sequencing were quantified by ddPCR. The analysis confirmed either the absence of detectable *Bacillus* or a concentration below 10^2^ spores × mL^−1^ (or g^−1^) (data not shown). Only tea and herbal infusion products represented an exception, with some samples showing *Bacillus* levels above 10^2^ spores × mL^−1^ (or g^−1^) (data not shown), despite the absence of a visible band on the agarose gel (Supplementary Figure S3).

**Figure 3:**
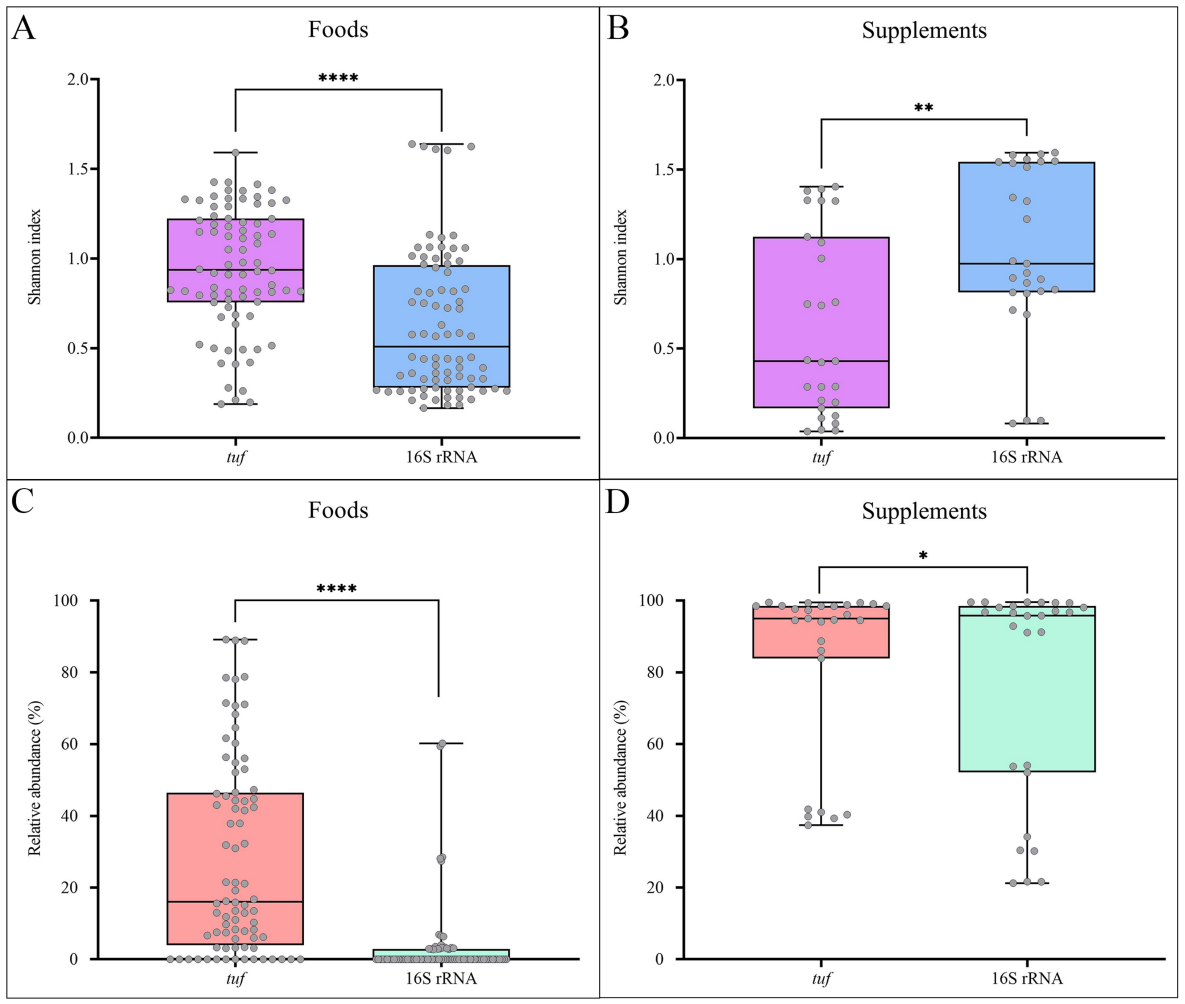
Alpha-diversity based on Shannon index representing *Bacillus* and closely related genera communities in **(A)** Food and **(B)** Supplement categories (Wilcoxon signed-rank test; ^****^: *p*-value < 0.0001; ^**^: *p*-value < 0.01). Box plots representing the cumulative relative abundance of *Bacillus* and closely related genera in **(C)** Food and **(D) S**upplement products (Wilcoxon signed-rank test; ^****^: *p*-value < 0.0001; ^*^: *p*-value < 0.05).

On average, *tuf* and 16S rRNA sequencing generated 212,358 and 122,398 reads per sample, respectively (Supplementary Tables S6, S7). Regarding the food products category, alpha diversity analysis revealed differences between the two sequencing approaches, when focusing on the *Bacillus* and closely related genera community. More specifically, *tuf*-based sequencing results revealed a significantly higher bacterial diversity (*p*-value < 0.0001), expressed by the Shannon index, compared to the 16S rRNA data (Figure 3A). In contrast, for the supplements category (Figure 3B), the 16S rRNA sequencing approach produced significantly higher Shannon index values (*p*-value < 0.01) than those obtained by the *tuf* gene-based method.

When analysing the cumulative relative abundance of the *Bacillus* and closely related genera in tested samples, significant differences were observed for both product categories between *tuf*- and 16S rRNA-based ONT methods (Figures 3C,D). These differences were evaluated using the Wilcoxon signed-rank test, applied to matched pairs of samples processed with both approaches (foods: *p*-value < 0.0001; supplements: *p*-value < 0.05).

**Figure 4:**
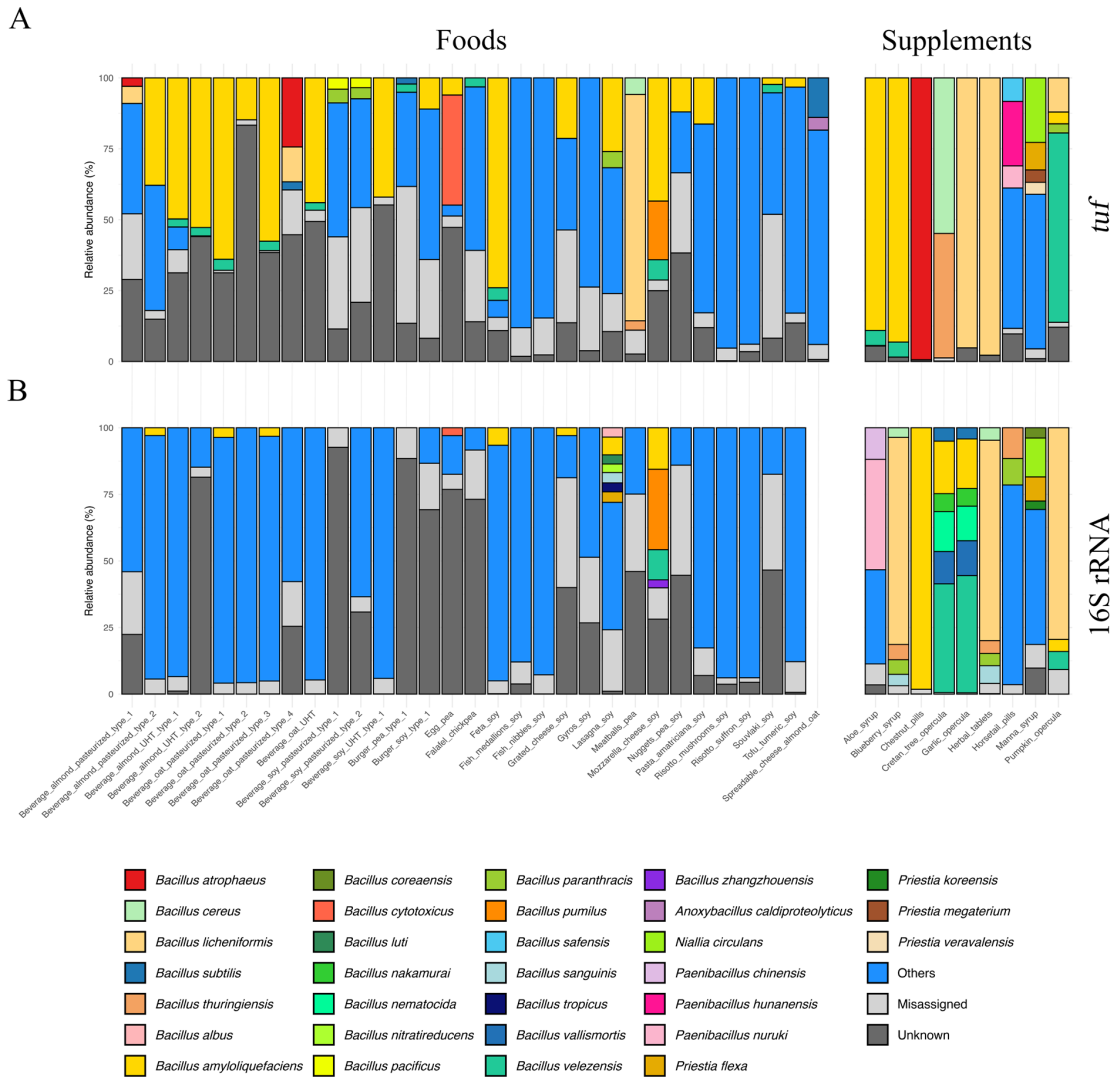
Stacked bar charts representing the *Bacillus* and closely related genera community in commercial plant-based food and supplements products ONT-sequenced with **(A)** *tuf* and **(B)** 16S rRNA genes. All the identified taxa in each replicate whose relative abundance is lower than 2.5% are collapsed into the “Misassigned” group; unclassified species are grouped as “Unknown”; non-*Bacillus* and non-closely related genera are grouped as “Others.” The ONT 16S rRNA sample “Spreadable_cheese_almond_oat” was excluded from the dataset after the rarefaction step.

Figure 4 shows stacked bar plots of the samples, grouped by target gene and product category, illustrating the relative abundances distribution of *Bacillus* and closely related genera across the two amplification methods and sample types. Specifically, *tuf*-based sequencing (Figure 4A) detected *Bacillus* and closely related genera in 35 out of 40 products, with *B. amyloliquefaciens*, *B. velezensis,* and *B. licheniformis* being the most frequent species, detected in 21, 13, and 6 products, respectively. Members of the *B. cereus* group (comprehending *B. cereus, B. thuringiensis, B. cytotoxicus, B. pacificus*, and *B. paranthracis*) were found in seven products, while other *Bacillaceae* such as *Anoxybacillus* and *Paenibacillus* were also identified in one product. Additional species included *B. atrophaeus*, *B. subtilis*, and several others, were found, as shown in Figure 4A and Supplementary Tables S8, S9. Genera other than *Bacillus* and closely related genera were also detected, with relative abundances ranging from 3 to 95% (Supplementary Figure S4). In the remaining 5 out of 40 products, *Bacillus* or closely related genera were not detected; instead, reads were assigned to other bacterial taxa, mainly including *Enterococcus*, *Carnobacterium,* and *Macrococcoides* genera (Supplementary Figure S4).

On the other hand, 16S rRNA gene sequencing results (Figure 4B) indicated a lower incidence of *Bacillus* and closely related genera among tested products when compared to *tuf* gene analysis; indeed, the *Bacillus* genus was found in 17 out of 39 products (one product was excluded after the rarefaction step: “Spreadable_cheese_almond_oat”), including *Paenibacillus* in one product, while An*oxybacillus* was not detected at all. Species belonging to the *B. subtilis* group and *B. cereus* group were detected in 15 and 5 products, respectively. Additional species detected at lower frequency included *B. nakamurai*, *B. nematocida*, and *P. flexa,* as shown in Figure 4B and Supplementary Tables S8, S9.

### 3.3 Identification of *Bacillus* spp. isolates by culture-based analysis

Out of 72 commercial products, *Bacillus* and closely related genera colonies were successfully isolated from 46 samples after enrichment on PEMBA plates. The isolation rates by product category were as follows: in 18% of the beverages, in 79% of the solid plant-based foods, and in 78% of the supplements (Supplementary Tables S8, S9). No bacterial growth was observed in the remaining 26 products. From the 46 culture-positive products, a total of 64 bacterial colonies were subjected to Sanger sequencing of the 16S rRNA gene. Among these, 48 colonies were confirmed as *Bacillus* spp., while 16 colonies belonged to closely related genera, including *Paenibacillus* (7 colonies), *Lysinibacillus* (3 colonies), *Priestia* (3 colonies), *Niallia* (2 colonies), and *Brevibacillus* (1 colony). Notably, 16 colonies from 15 different products were identified as belonging to the *B. cereus* group, whereas 11 colonies from 19 different products were included in the *B. subtilis* group.

### 3.4 ONT- and culture-based analyses comparison

**Figure 5:**
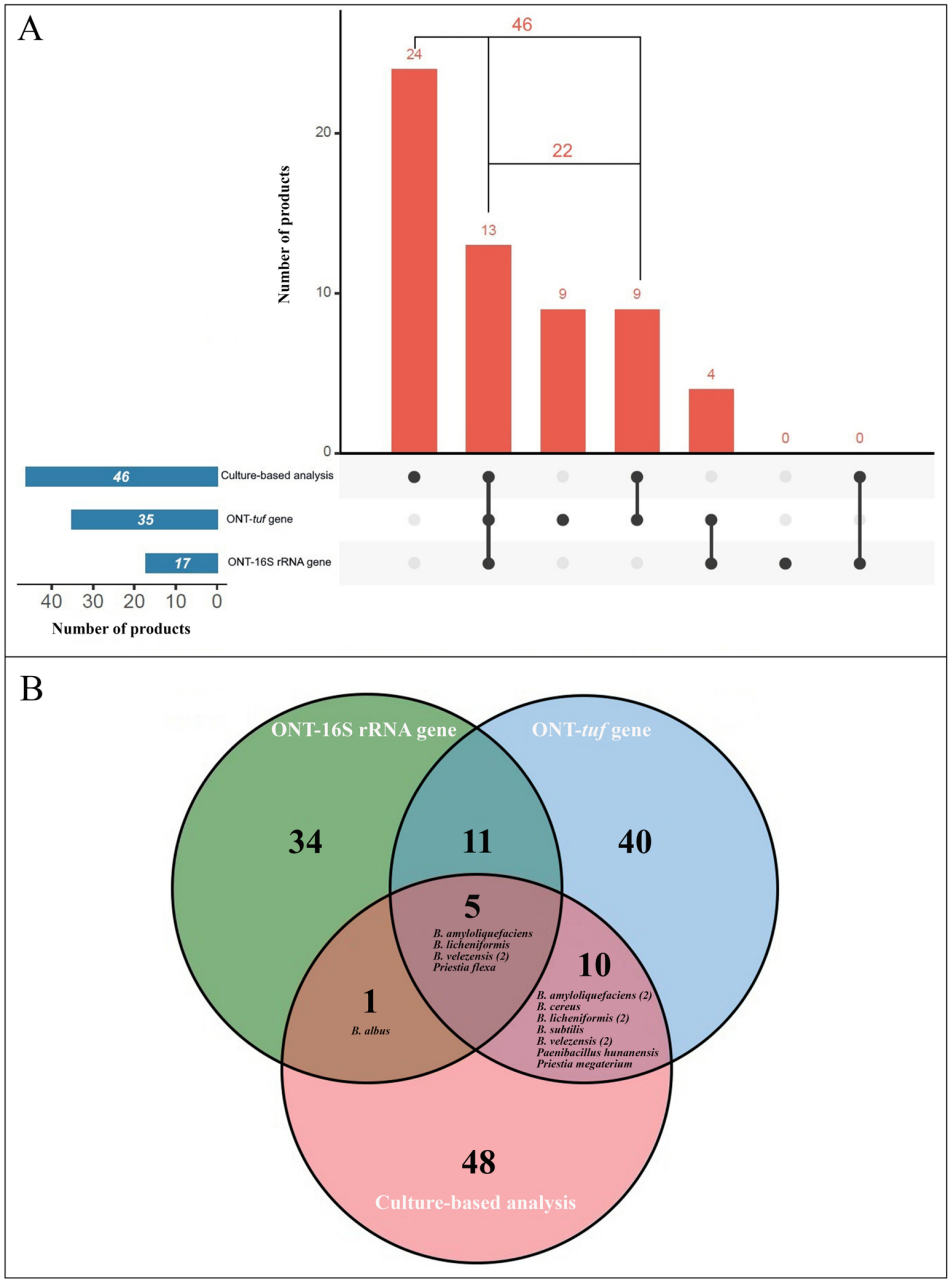
Comparison of taxonomic identifications obtained using ONT-*tuf* gene sequencing, ONT-16S rRNA gene sequencing, and conventional culture-based methods. **(A)** UpSet plot showing the presence and absence of *Bacillus* and closely related genera detected by each technique. **(B)** Venn diagram illustrating shared and unique *Bacillus* species identified across the three approaches.

Among the 46 commercial plant-based products positive for *Bacillus* spp. and closely related genera by culture-based analysis, *tuf* gene sequencing confirmed the presence in 22 out of 46 culture-positive cases, while 16S rRNA gene sequencing confirmed it in 13 products. Notably, in 9 samples where both culture-based and *tuf* gene analysis detected *Bacillus* spp., 16S rRNA gene sequencing failed to identify their presence (Figure 5A; Supplementary Tables S8, S9). Conversely, all the products that resulted positive by both culture-based analysis and 16S rRNA gene sequencing, were also confirmed by *tuf*-gene analysis.

Regarding the taxonomic assignment of ONT sequences, 15 *Bacillus* species identified by *tuf* gene analysis matched the culture-based identification in 12 products, whereas 6 matching *Bacillus* species were identified by 16S rRNA gene sequencing in 5 products (Figure 5B; Supplementary Tables S8, S9).

## 4 Discussion

The possible contamination and proliferation of *Bacillus* spores in the fast-growing category of plant-based food products has already been described in numerous studies (Brown and Jiang, 2008; Dlugaszewska et al., 2019; Tóth et al., 2021; Bartula et al., 2023; Kyrylenko et al., 2023; Roch et al., 2024; Barmettler et al., 2025). However, the development of rapid and accurate methods to detect and identify *Bacillus* species in foodstuffs has been increasingly emphasized as a crucial need in food safety research (Lin et al., 2023). Accordingly, the objective of this study was to implement an NGS-based approach to detect and identify *Bacillus* spp. in plant-based products. In fact, traditional approaches typically involve culture steps that are both cost-effective and time-consuming (Saravanan et al., 2021). In addition, most of the classical microbiological approaches require an enrichment step prior to DNA extraction, necessary to increase extraction yield. However, this step can introduce bias by favoring the growth of one species predominate over another, as well as hindering the quantitative determination of species abundance and limiting the subsequent sequencing results (Piotrowska-Cyplik et al., 2017; Duthoo et al., 2022).

To overcome this, in this study, we implemented a direct DNA extraction protocol based on a combination of pre-processing of the food matrix by enzymatic digestion and mechanical cell lysis with bead beating, to maximize the recovery of *Bacillus* spore DNA from the food matrix. ddPCR measurements performed on inoculated food products allowed quantification of spores down to 10^1^ spores × mL^−1^ (or g^−1^) in all seven representative samples tested (protein-rich, fat-rich, starch-rich, polyphenols-rich products). This result confirmed the efficiency and robustness of the protocol in terms of DNA yield. Overall, the DNA extraction protocol performed quite better in liquid as compared to solid plant-based food, probably because of the higher complexity of the food matrix and/or presence of inhibitors. However, as mentioned, plant-based food products and supplements display high heterogeneity in terms of nutritional composition and physical characteristics; conditions may vary considerably not only between different food categories, but also across different products within the same group. Direct DNA isolation protocols can thus provide different results concerning DNA concentration, yield, and contaminant carryover, and may require further tailoring to account for the specific features of a given sample. Importantly, the slightly reduced recovery in herbal tea infusion further supports the presence of polyphenolic PCR inhibitors. While ddPCR quantification was only slightly affected by these inhibitors, thanks to the high dilution of the template across droplets, conventional PCR is likely more susceptible (Dingle et al., 2013; Whale et al., 2016). The observed lack of amplification by conventional PCR in some commercial herbal tea infusions may, therefore, be explained by matrix effects. Future studies should further explore the optimization of DNA extraction protocols for challenging matrices such as herbal teas, which are typically rich in polyphenols, polysaccharides, and other secondary plant metabolites known to interfere with nucleic acid isolation and enzymatic amplification (Wilson, 1997; Schrader et al., 2012). A more systematic evaluation of pre-treatment steps—such as the inclusion of binding resins, polyvinylpyrrolidone (PVP), or additional purification phases—could help mitigate the inhibitory effects of these compounds and improve DNA recovery efficiency.

The m-tufGP *in silico* evaluation revealed a clear trade-off between coverage and selectivity as the mismatch tolerance increased. At 0–1 mismatch per primer, sensitivity ranged from 82.9 to 96.6% and specificity remained high (≥ 99.0%), with precision (PPV) values above 76.2%, indicating reliable discrimination of *Bacillus* sequences. Allowing up to two mismatches reduced precision (PPV) to 42.3%, reflecting the inclusion of additional non-target sequences. At three mismatches, precision (PPV) dropped markedly from 42.3 to 10.7%, respectively, highlighting an increased risk of false-positive amplification. However, direct comparison with other genus-specific studies should be approached with caution, as apparent differences in sensitivity or specificity often result from methodological variability—such as database composition, target-gene heterogeneity, and mismatch evaluation criteria—rather than intrinsic primer efficiency (Henriques et al., 2012; Mancabelli et al., 2020).

In this study, a minimum concentration of 10^2^ spores × mL^−1^ (or g^−1^) was required to generate sufficient amplicon yield for ONT sequencing of the *tuf* and 16S rRNA genes. This threshold may represent a limitation when analyzing naturally contaminated products, in which *Bacillus* concentrations can be lower. This constraint could be mitigated by increasing the processed sample volume or by applying an enrichment or concentration step prior to DNA extraction to enhance sensitivity while minimizing potential compositional biases.

While European regulations do not specify any food safety criteria for *B. cereus*, studies indicate that more than 100 CFU/g of *B. cereus* in food can lead to foodborne infection (Ghelardi et al., 2002; Glasset et al., 2016). Regarding the ONT *tuf*-gene sequencing and the use of a *tuf* custom database (i.e., BacTufDB) to uncover the bacterial consortia in a sample, the simple mock community including five equimolar DNA of *Bacillus* spp. was correctly identified, with good discrimination of all the five highly related *Bacillus* species considered. The same was achieved with the 16S rRNA gene. However, considering the five *Bacillus* species included in the mock community, *tuf-*based sequencing more accurately reflected the expected proportions (as indicated by higher Bray–Curtis similarity values), demonstrating improved performance within the targeted genus. Despite the different percentages of “Misassigned” and “Unknown” sequences that could be intrinsically due to the use of different databases (*tuf* and 16S rRNA), a lower relative abundance of these two features was achieved with the *tuf*-based strategy.

When sequencing the four inoculated food samples, both *tuf* and 16S rRNA sequencing detected the five *Bacillus* species present in the spiked food matrices; as observed in the mock communities, the *tuf*-based assay more accurately reproduced their actual relative abundances. Even in mock and spiked samples inoculated with known *Bacillus* species, a residual proportion of “Unknown” reads was detected, likely resulting from PCR-derived artifacts or analytical variability accumulated across processing steps, potentially affecting read classification accuracy and taxonomic assignment.

A total of 72 products were tested by *tuf* and 16S rRNA gene amplification, and samples yielding visible PCR products were subsequently sequenced using ONT. The same 72 products were tested by culture-based analysis. Differently from previous studies (Roch et al., 2024; Barmettler et al., 2025), which focused solely on meat analogues, this study considers a broader range of products, including UHT and pasteurized plant-based beverages, meat and fish analogues, alternative cheeses, vegan pasta, and plant-based food supplements.

Among the 72 samples tested, species of *Bacillus* and closely related genera were detected in 46 samples by the cultivation method, while in 35 and 17 products, with ONT sequencing, using the *tuf* and the 16S rRNA marker genes, respectively. These results suggest a higher sensitivity of the culture-based approach, likely due to the enrichment step inherent in such method. This step may have contributed to improve detection by lowering the threshold level relative to the ONT sequencing workflow (previously established at 10^2^ spores × mL^−1^ or g^−1^), in part by promoting the formation of vegetative cells that are notably easier to lyse. Additionally, it may have helped to reduce interference from the food matrix. However, it is important to highlight that species-level identification relying on full-length 16S rRNA gene is not sufficiently discriminative for closely related species such as the ones belonging to the *B. cereus* and *B. subtilis* groups. These taxa share highly conserved 16S rRNA sequences (> 99% identity), making it difficult to resolve them accurately at the species level (Blackwood et al., 2004; Lee et al., 2022). Consequently, the taxonomic assignments obtained should be considered indicative of the genus group affiliation, even if the species level was reported for the purpose of comparison with the ONT-based analyses. Moreover, it is important to stress that the enrichment step may also introduce biases in biological representativeness, as it can selectively support the growth of individual *Bacillus* species. Additionally, out of the 26 culture-positive samples for which no amplification product was obtained either for *tuf* or 16S rRNA genes, the majority belonged to the herbal infusion category. This finding is consistent with the results obtained during the extended ddPCR validation, where the herbal tea infusion showed slightly lower DNA quantification compared to the other matrices. This suggests that polyphenol-rich products may contain inhibitory substances that reduce DNA recovery and strongly affect amplification efficiency. While the partitioning of the reaction in ddPCR minimizes the effect of such inhibitors (Dingle et al., 2013), conventional PCR and long-read sequencing methods like ONT remain more sensitive to inhibition. This could explain why most herbal tea infusions tested were culture-positive for *Bacillus* spp. but did not yield amplification products, further supporting the need for targeted optimization of the extraction protocol for these matrices.

When focusing on *Bacillus*-positive samples as confirmed by culture-based analysis, *tuf* gene sequencing provided concordant positive results in about 50% of the samples tested, compared to 30% with the 16S rRNA-based analysis. These results suggest that the m-tufGP primers performed better than the 16S rRNA gene universal primers in detecting *Bacillus* in food products where, unlike in artificially contaminated products, *Bacillus* may occur in lower proportions as compared to other microbial populations. However, a value of 50% indicates that a portion of the positive samples were not detected using the *tuf-*based ONT sequencing approach, resulting in an underestimation of the samples contaminated with *Bacillus*. This certainly represents a major limitation of the proposed approach, which will require further investigation. Remarkably, the culture-based method benefited from an enrichment step prior to bacterial isolation, which was not applied for gene sequencing. Future testing will be warranted to assess the suitability of including an enrichment step before DNA extraction to increase the detection rate of positive samples using the *tuf-*based sequencing approach. Importantly, there were no instances where *Bacillus* was detected by both 16S rRNA sequencing and culture-based methods but missed by *tuf* gene analysis. Remarkably, we observed a subset (9 out of 72 products, mainly including heat-treated beverages) of culture-negative and 16S rRNA negative samples in which *tuf* gene analysis found *Bacillus* species. This finding may indicate the presence of free DNA or DNA from non-culturable cells, which can persist in heat-treated or otherwise processed products and remain detectable by PCR. The discrepancy observed between *tuf* and 16S amplicon sequencing results could be attributed to differences in primer specificity toward *Bacillus* sequences. Regarding the taxonomic assignment of ONT sequences, *tuf*-based sequencing provided greater consistency in *Bacillus* species identification with cultivation results, likely due to its higher resolution capacity within the *Bacillus* genus (Caamaño-Antelo et al., 2015), as previously reported in other genera (Naser et al., 2007; Li et al., 2012; Huang et al., 2014; McMurray et al., 2016; Pourahmad, 2025).

When comparing the two ONT approaches across both sample categories (food and supplement products), the Wilcoxon signed-rank test revealed a statistically significant difference in the cumulative relative abundance of *Bacillus* and closely related genera between the *tuf*- and 16S rRNA-based profiles. The higher *Bacillus* diversity observed with the *tuf*-based sequencing in food products likely reflects the marker’s primer design, which selectively enriches *Bacillus* sequences and thus provides deeper coverage within this genus. An exception within the food category was represented by the *Lasagna_soy* product, which showed a higher *Bacillus* diversity with the 16S rRNA gene marker compared to the *tuf*-based profiling, as also observed in the supplement product category. This pattern may be explained by the higher native predominance of *Bacillus* spp. in these samples, together with the broader taxonomic coverage of the 16S rRNA primers, which can detect a wider range of related species.

In 11 out of 40 products sequenced using the *tuf* gene, over 50% relative abundance of the assigned species did not belong to *Bacillus* and closely related genera. This could be due to the low abundance of the target *Bacillus* DNA, coupled with the presence of non-target DNA from other foodborne bacteria, since mispriming events can occur when template concentrations are low, leading to non-specific amplification (Damavandi et al., 2021). 16S rRNA gene analysis, instead, displayed consistently higher relative abundances of unassigned taxa compared to the *tuf* gene.

Coupled with the BacTufDB database for taxonomic classification, MinION *tuf*-based sequencing effectively identified and differentiated most *Bacillus* species. However, it is important to note that the applied methodology relies on a closed-reference mapping approach; therefore, closely related species, such as those belonging to either the *B. cereus* or *B. subtilis* groups, require further confirmation using additional orthogonal genetic markers (e.g., *rpoB*, *gyrB*) or whole-genome sequencing (WGS) (Liu et al., 2015; Wang et al., 2007; Xu et al., 2023).

Despite 16S rRNA-based sequencing has been considered the reference method for the identification of microorganisms in foodstuffs, this gene can fail a proper classification of *Bacillus* species, especially when using amplicons of the V3-V4 region (Strube, 2021). Full-length 16S rRNA sequencing can provide a better microbial species-level resolution (Kerkhof et al., 2017); however, for *Bacillus* genus, it has been previously proven the ability of *tuf* gene to better distinguish closely related species belonging to the *Bacillus subtilis* group (Caamaño-Antelo et al., 2015), that can be specifically found in plant-based food products (Kyrylenko et al., 2023). Furthermore, it has been also stressed the need for a customized *tuf* gene database to improve the resolution of species identification (Strube, 2021; Xu et al., 2023). The 72 samples analyzed in this study do not represent an extremely large number of samples, although they do provide a representative sample of the main categories of plant-based products. Therefore, future studies could apply the methodology presented in this study to track a more comprehensive number of samples. Nevertheless, *tuf-*gene sequencing with MinION confirmed *Bacillus* and closely related genera as frequently present in the plant-based supply chain (35 out of 40 of the sequenced samples) and highlighted *Bacillus cereus* group (7 out of 40), posing a potential health risk to consumers. Although in some of the tested products the ONT 16S rRNA gene-based analysis was able to distinguish a wider range of *Bacillus* species, the ONT *tuf* analysis enabled the detection of *Bacillus* spp. in 18 products (out of 35) where the 16S rRNA analysis failed to identify them.

In conclusion, this study presents an optimized workflow aimed at improving the efficient recovery and identification of *Bacillus* sub-populations in plant-based food products. Based on the results, it can be concluded that the *tuf* gene can be an accurate alternative to the 16S rRNA gene-based analysis for the detection and taxonomic identification of *Bacillus* and closely related genera in food products, with the advantage of being able to better highlight the presence of contaminating *Bacillus* species in the products. Based on the proposed workflow, the entire process, from DNA extraction to ONT real-time data acquisition, basecalling and bioinformatic analysis, can be completed within 24 h per sample, offering a significant time advantage over traditional culture-based methods, that require extended incubation and confirmation steps. In contrast to targeted molecular techniques such as quantitative PCR, which provide rapid results but are confined to specific organisms, non-targeted approaches, including gene sequencing, allow for an unbiased and comprehensive characterization of microbial communities. Overall, the implemented approach may enhance the efficiency of food safety assessments and control applications.

## Data Availability

The ONT sequencing reads were deposited in the NCBI Sequence Read Archive (SRA), under the BioProject PRJNA1158730 (Biosamples: SAMN43546484-SAMN43546573; SAMN47172496-SAMN47172535; SAMN47177166-SAMN47177271; SAMN47178037-SAMN47178138).

## Footnotes

1
https://www.ncbi.nlm.nih.gov/tools/primer-blast/

2
https://rrndb.umms.med.umich.edu/

3
http://weblogo.berkeley.edu/

4
https://github.com/wdecoster/NanoPlot/tree/v1.42.0
